# DeepEnzyme: a robust deep learning model for improved enzyme turnover number prediction by utilizing features of protein 3D-structures

**DOI:** 10.1093/bib/bbae409

**Published:** 2024-08-20

**Authors:** Tong Wang, Guangming Xiang, Siwei He, Liyun Su, Yuguang Wang, Xuefeng Yan, Hongzhong Lu

**Affiliations:** State Key Laboratory of Microbial Metabolism, School of Life Science and Biotechnology, Shanghai Jiao Tong University, 800 Dongchuan RD. Minhang District, Shanghai 200240, China; College of Science, Chongqing University of Technology, 69 Hongguang Avenue, Banan District, Chongqing 400054, China; State Key Laboratory of Microbial Metabolism, School of Life Science and Biotechnology, Shanghai Jiao Tong University, 800 Dongchuan RD. Minhang District, Shanghai 200240, China; State Key Laboratory of Microbial Metabolism, School of Life Science and Biotechnology, Shanghai Jiao Tong University, 800 Dongchuan RD. Minhang District, Shanghai 200240, China; College of Science, Chongqing University of Technology, 69 Hongguang Avenue, Banan District, Chongqing 400054, China; Institute of Natural Sciences, School of Mathematical Sciences, Zhangjiang Institute of Advanced Study, Shanghai Jiao Tong University, 800 Dongchuan RD. Minhang District, Shanghai 200240, China; Shanghai Artificial Intelligence Laboratory, 701 Yunjin Road, Xuhui District, Shanghai 200237, China; Key Laboratory of Smart Manufacturing in Energy Chemical Process, Ministry of Education, East China University of Science and Technology, 130 Meilong Road, Xuhui District, Shanghai 200237, China; State Key Laboratory of Bioreactor Engineering, East China University of Science and Technology, 130 Meilong Road, Xuhui District, Shanghai 200237, China; State Key Laboratory of Microbial Metabolism, School of Life Science and Biotechnology, Shanghai Jiao Tong University, 800 Dongchuan RD. Minhang District, Shanghai 200240, China

**Keywords:** enzyme turnover number, deep learning, protein 3D-structure, graph convolutional network

## Abstract

Turnover numbers (*k*_cat_), which indicate an enzyme's catalytic efficiency, have a wide range of applications in fields including protein engineering and synthetic biology. Experimentally measuring the enzymes' *k*_cat_ is always time-consuming. Recently, the prediction of *k*_cat_ using deep learning models has mitigated this problem. However, the accuracy and robustness in *k*_cat_ prediction still needs to be improved significantly, particularly when dealing with enzymes with low sequence similarity compared to those within the training dataset. Herein, we present DeepEnzyme, a cutting-edge deep learning model that combines the most recent Transformer and Graph Convolutional Network (GCN) to capture the information of both the sequence and 3D-structure of a protein. To improve the prediction accuracy, DeepEnzyme was trained by leveraging the integrated features from both sequences and 3D-structures. Consequently, DeepEnzyme exhibits remarkable robustness when processing enzymes with low sequence similarity compared to those in the training dataset by utilizing additional features from high-quality protein 3D-structures. DeepEnzyme also makes it possible to evaluate how point mutations affect the catalytic activity of the enzyme, which helps identify residue sites that are crucial for the catalytic function. In summary, DeepEnzyme represents a pioneering effort in predicting enzymes' *k*_cat_ values with improved accuracy and robustness compared to previous algorithms. This advancement will significantly contribute to our comprehension of enzyme function and its evolutionary patterns across species.

## Introduction

The enzyme turnover number (*k*_cat_) represents the maximum number of substrate molecules that an enzyme can convert into a product per unit time under saturating conditions [1, 2]. Presently, accurately predicting *k*_cat_ has become imperative for various applications, including protein engineering and enzyme design [3]. Additionally, estimating enzyme *k*_cat_ values at genome-scale is crucial for constructing sophisticated metabolic models to capture the correlations between genotypes and phenotypes [4, 5]. Despite the abundant *k*_cat_ values available in databases [6, 7], the total number of enzymes with experimentally determined *k*_cat_ remains substantially smaller than the vast number of sequenced proteins [8].

Recently, it witnessed the breakthroughs in development of various advanced deep learning models, which make it possible to predict protein structure [9], protein function [10], and gene expression level [11] only from primary sequence features. Heckmann et al. [12] firstly utilized machine learning models, i.e. the linear elastic net, the decision-tree-based random forest model, to infer the enzyme *k*_cat_ for the model organism *Escherichia coli*. However, its application is only suitable for *E. coli*. Subsequently, the deep learning model-DLKcat, proposed by Li et al. [13], significantly broadens the scope of *k*_cat_ prediction for nearly all sequenced enzymes with catalyzed substrates. By incorporating features from both the protein sequence and its substrate, DLKcat facilitated high-throughput prediction of enzyme *k*_cat_ and the automatic generation of genome-scale *k*_cat_ profiles. To further enhance the performances of DLKcat, TurNuP takes into account the fingerprint features from both substrates and products within a biochemical reaction [14]. Trained by a refined dataset, TurNuP outperforms DLKcat in predicting *k*_cat_ for enzymes with low sequence similarity compared to those in the training dataset. However, there remains room for improvement in the prediction accuracy of TurNuP.

To a larger extent, the function of a protein is determined by its 3D-structure [15]. The structural data provide crucial insights into the spatial arrangement of pivotal functional residues and the interactions between the enzyme and its substrate [16, 17]. The composition of amino acids and the distinct folding pattern within the protein structure can significantly influence the accessibility of active sites, substrate binding sites, and the overall stability of the enzyme [18]. These factors collectively impact enzyme function and its catalytic efficiency. Consequently, features derived from protein 3D-structures have garnered special attention in the development of advanced deep learning models [19–21]. Recent breakthroughs in protein structure prediction [9, 22–24], have substantially reduced the cost in acquiring high-quality protein structures. Thus, it is feasible to build large-scale structure datasets for the training of advanced deep learning models. However, the invaluable protein 3D structural information spanning various species has not been systematically utilized in enzyme *k*_cat_ prediction.

We present a novel deep learning model, named as DeepEnzyme, to enhance the prediction accuracy of enzyme *k*_cat_ based on protein sequence and 3D-structures. DeepEnzyme combines Graph Convolutional Network (GCN) [25], a proven approach for extracting structural properties from proteins [26, 27], with Transformers [28], which have demonstrated exceptional performance in protein language modeling [29, 30]. This fusion allows DeepEnzyme to extract both structure and sequence features from enzymes and substrates, thus provides rich biochemical information for learning enzyme protein in the catalysis. Compared with existing models, DeepEnzyme exhibits improved accuracy and robustness in predicting *k*_cat_ values. Therefore, DeepEnzyme has the potential to expedite the functional analysis of previously unstudied enzymes and provide opportunities for the rational optimization of enzyme activities in conjunction with advanced molecular technologies.

## Results

### Framework of a novel deep learning model for predicting *k*_cat_

**Figure 1:**
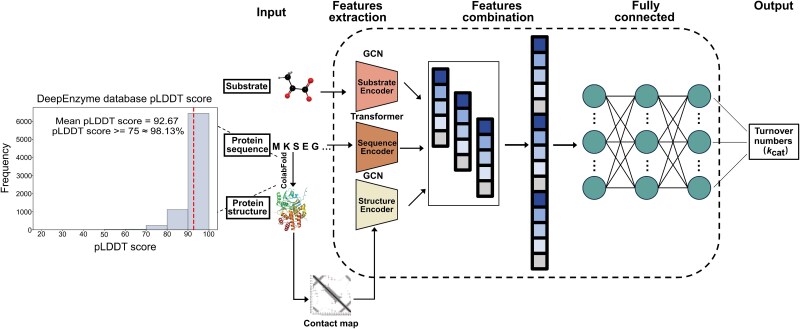
**The framework of DeepEnzyme for *k*cat prediction.** DeepEnzyme integrates transformer and GCN models to distill features from both the enzyme and substrate for predicting *k*_cat_. GCN is employed to extract structural features based on protein 3D-structures and substrate adjacency matrices; transformer is utilized to extract sequence features from protein sequences.

The novel deep learning model for *k*_cat_ prediction, DeepEnzyme, was developed by integrating three layers of features extracted respectively from protein 1D-sequence, 3D-structure, and the related substrate, utilizing distinct computation modules (Methods, Fig. 1). Specifically, Transformer model was employed to extract features from the protein 1D sequence. Simultaneously, the 3D-structure underwent transformation into a contact map employing the ${C}_{\alpha }-{C}_{\alpha }\! < \!10\text{\AA} $ criteria. Subsequently, GCN was applied to extract structural features from the protein 3D-structures based on the contact map. Concurrently, the RDKit [31] was employed to extract crucial substrate features, including fingerprints and adjacency matrices, based on its SMILES formula. To delve deeper into substrate features, the fingerprints and adjacency matrices were further fed into a dedicated GCN module. Following this, all feature vectors extracted from both enzyme and substrate were merged in the subsequent phase. This process resulted in the creation of a comprehensive embedding vector, encapsulating features from both enzyme and substrate. Finally, the *k*_cat_ value was predicted employing a neural attention method based on the representation vectors.

Since a significant portion of enzymes in our dataset lacked experimentally determined 3D-structures, ColabFold [23] was utilized to predict the structures for all these proteins. The average predicted local distance difference test (pLDDT) score for all predicted protein 3D-structures in this study was 92.67 (Fig. 1), guaranteeing that the structures used for DeepEnzyme training are of high-quality.

### DeepEnzyme exhibits improved performance in *k*_cat_ prediction when taking protein 3D-structures into account

**Figure 2:**
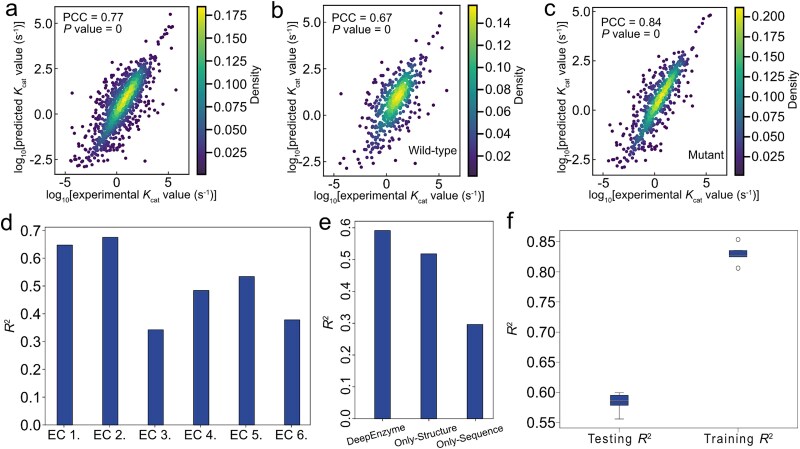
**Evaluation of DeepEnzyme performance in *k*cat prediction.** (a) the performance of DeepEnzyme on the test dataset was evaluated by the Pearson correlation coefficient (PCC) and *P* values calculated from the predicted and experimental *k*_cat_ values. (b-c) DeepEnzyme prediction performance on wild-type (b) and mutant (c) enzymes in the test dataset. (d) DeepEnzyme prediction performance for enzymes classified by different EC numbers in the test dataset. (e) the performance of the model with different types of datasets as input. DeepEnzyme: Enzyme sequence, enzyme structure, and substrate information are used as inputs; Only-structure: Substrate information and enzyme structure information as inputs; Only-sequence: Substrate and enzyme sequence as inputs. In (a)–(e) specific seed was adopted during the calculation. (f) Comparison in average coefficient of determination (R^2^) values for testing dataset and training dataset from five rounds of training.

Previously, it was demonstrated that the great similarity in protein sequences between the training, validation, and testing datasets can lead to overfitting and poor generalization of deep learning models in *k*_cat_ value prediction [14]. To mitigate this challenge, we preprocessed the data (Methods) to exclude highly similar sequences existing in the original DLKcat dataset [14, 32]. Consequently, out of the 16 838 distinct enzyme-substrate pairs, we carefully selected and retained 11 927 of all pairs, enabling us to reconstruct a dataset that could, to some extent, avoid the too high similarity in protein sequences used for model training. This preprocess of the enzymatic dataset allowed us to train and evaluate DeepEnzyme effectively. By mitigating the issue of sequence similarity and incorporating additional protein structure information, DeepEnzyme achieved a high Pearson correlation coefficient (PCC) value, close to 0.77 (Fig. 2a) on the test dataset after the dedicated training phase, indicating that it has a comparatively low prediction error.

To systematically evaluate the performance of DeepEnzyme, the predicted and measured *k_cat_* values for enzymes from diverse sources and classifications were compared thoroughly. Initially, enzymes were categorized into two main types: wild-type and mutant, based on their records in the public databases. The results indicated that, for mutant enzymes, the PCC was 0.84 (Fig. 2c), whereas for wild-type enzymes, it was only 0.67 (Fig. 2b). Notably, mutant enzymes constituted ~59% of all enzymes, suggesting that the prediction accuracy might be influenced by the quantity of enzymes from each group. Next, all enzymes were classified based on the first digit originated from the corresponding EC number. We found that DeepEnzyme exhibits distinct predictive capabilities for enzymes of varying EC numbers. For example, the R^2^ for enzymes from the EC 1 group and the EC 2 group was notably higher (Fig. 2d). This observation is consistent with the fact that the percentages of enzymes from these two groups in the dataset (37%, 26%) are significantly higher than the other enzyme groups, underscoring that the number of enzymes in each group could affect the model prediction capability. Furthermore, we compared the performance of DeepEnzyme using different types of datasets as input (Fig. 2e). It clearly showed that prediction accuracy was much increased by the addition of structural data. Lastly, to avoid the bias in the dataset sampling, the average R^2^ value on the testing dataset from five rounds of training was calculated, which achieved at 0.58 by DeepEnzyme, indicating the robustness of model prediction when using different test datasets (Fig. 2f). In summary, DeepEnzyme performs well in *k_cat_* prediction as a whole when taking features of protein 3D-structures into account though in some cases the prediction accuracy could be negatively affected by the related data size.

### DeepEnzyme has better performance in *k*_cat_ prediction compared with existing models

**Figure 3:**
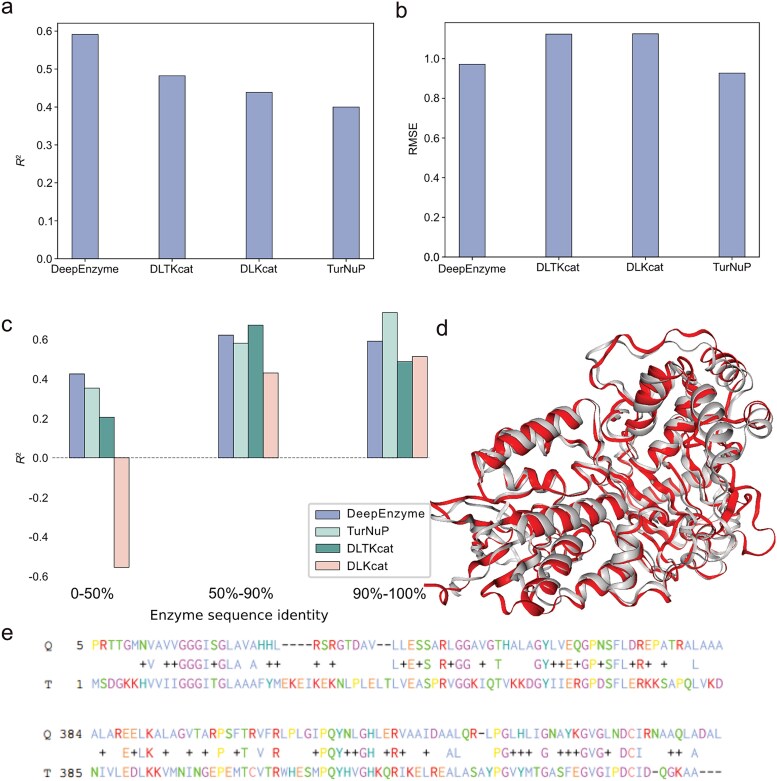
**Improved performances of DeepEnzyme in *k*cat prediction compared to existing models, even for protein sequences in the test dataset exhibiting lower similarity compared to those in the training dataset.** (a) Comparison of R^2^ values on the test dataset for different models. (b) Comparison of RMSE values on the test dataset for different models. (c) Comparison of R^2^ in *k*_cat_ value prediction for enzymes in the test dataset at different levels of sequence similarity by DeepEnzyme, TurNuP, DLKcat, and DLTKcat. (d) Two enzymes from *Myxococcus xanthus* and *Bacillus subtilis*, both with EC numbers 1.3.3.4, are highly similar in protein 3D-structure (TM-score = 0.8762), gray for enzyme from *M. xanthus* and red for enzyme from *B. subtilis*. (e) the similarity of the amino acid sequences for the above two enzymes is 27% (Q for enzyme from *M. xanthus*, T for enzyme from *B. subtilis*).

To comprehensively compare DeepEnzyme with existing *k*_cat_ prediction tools, three of the latest publicly available models with accessible scripts, TurNuP [14], DLKcat [13], and DLTKcat [33], were selected and evaluated together with DeepEnzyme. By contrast, when utilizing protein sequence, 3D-structure and substrate as input, DeepEnzyme exhibited significantly improved prediction accuracy (${R}^2\approx 0.6$) compared to TurNuP, DLKcat and DLTKcat (Fig. 3a). In addition, the RMSE value of DeepEnzyme was 0.95, which was lower than that of DLKcat and DLTKcat but slightly higher than TurNuP (Fig. 3b). The lower RMSE value in TurNuP may be due to the fact that the authors omitted the enzyme-catalyzed reactions with exceptionally low or high *k*_cat_ values during the training phase, resulting in a drop in the model’s final RMSE value [14]. Overall, it could be concluded that DeepEnzyme performs better in *k*_cat_ prediction when utilizing protein structure as input.

The sequence similarity has a significant impact on deep learning model performance in predicting enzyme function, particularly in predicting the substrates catalyzed by individual enzymes [32]. Notably, the prediction accuracy can significantly diminish when the protein sequences in the test dataset exhibit lower similarity to those in the training dataset. To assess the performance of DeepEnzyme at various levels of sequence similarity, we employed the MMseqs2 [34] to compute sequence similarity between the test and training datasets. Subsequently, based on the calculated similarity, the test dataset was divided into three distinct groups: 0–50%, 50–90%, and 90–100%. We then calculated the R^2^ value for each group (Fig. 3c). Remarkably, DeepEnzyme showed an impressive R^2^ value even when the sequence similarity spanned from 0 to 50%, demonstrating its robustness in *k*_cat_ prediction under low sequence similarity situations.

In a detailed comparison of the predicted performance of TurNuP, DLKcat, DLTKcat, and DeepEnzyme on enzymes with varying levels of sequence similarity, it was observed that DLKcat and DLTKcat experienced a substantial change in R^2^, whereas the corresponding R^2^ remained relatively stable for DeepEnzyme (Fig. 3c). It may be due to the fact that, during model training phase, DeepEnzyme learnt useful features of protein 3D-structures, which are closely correlated to the enzyme function. In reality, homologous enzymes can exhibit higher conservation in 3D-structures while displaying divergent evolutionary paths in terms of sequences. As one of the typical examples, there are two protein sequences in our datasets having the same EC number − 1.3.3.4, the final common enzyme in the chlorophyll and heme biosynthesis, from different species (*Myxococcus xanthus* and *Bacillus subtilis*). Calculated by Foldseek [35] and US-align [36], it revealed that the structural similarity between those two enzymes is as high as 0.88 (Fig. 3d), although their sequence similarity is only 0.27 (Fig. 3e). Together, our findings demonstrate DeepEnzyme’s capacity to successfully use 3D structural information for enhanced prediction, while also showcasing robustness in prediction accuracy over a wide range of sequence similarities.

### Evaluation of DeepEnzyme’s prediction capability using enzyme saturation mutagenesis datasets

**Figure 4:**
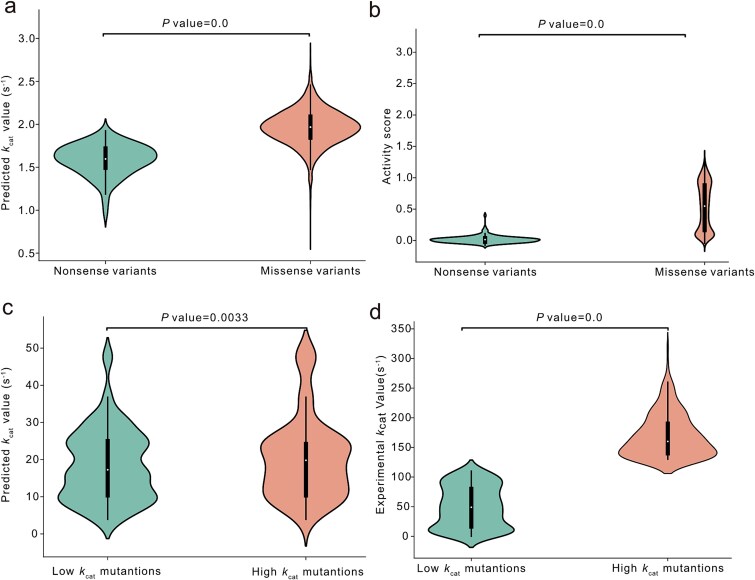
**Analysis of the prediction ability of DeepEnzyme for two enzymes with saturation mutagenesis datasets.** (a) Comparison of predicted results for different CYP2C9 variants: Red for missense variants, green for nonsense variants. (b) Comparison of experimental activity score for different CYP2C9 variants: Red for missense variants, green for nonsense variants [40]. (c) Comparison of predicted *k*_cat_ values for different PafA mutations: Green for low *k*_cat_ mutations, red for high *k*_cat_ mutations [41]. (d) Comparison of experimentally measured *k*_cat_ values for different PafA mutations: Green for low *k*_cat_ mutations, red for high *k*_cat_ mutations [41].

To validate the capability of DeepEnzyme in predicting the impact of mutations on the catalytic efficiency of enzymes, we firstly compared the predicted *k*_cat_ from DeepEnzyme with the high-throughput experimental datasets for CYP2C9, a critical enzyme in drug metabolism and personalized therapy [37–40], which encompasses more than 6500 mutated sequences with measured enzyme activity [40]. Based on the enzyme activity, all variants of CYP2C9 were categorized into three groups: missense variants, synonymous variants, and nonsense variants. We utilized the well-trained DeepEnzyme to predict and compare the *k*_cat_ values of missense variants and nonsense variants (no comparison was conducted for synonymous variants as they do not undergo sequence alterations). The result clearly indicates that the predicted *k*_cat_ values for nonsense variants (median: *k*_cat_ = 1.60s^−1^) were lower than those of missense variants (median: *k*_cat_ = 1.97 s^−1^, *P* value = 1.6 × 10^−94^) (Fig. 4a). Interestingly, such a result is consistent with the experimental evidence in which the nonsense variants have a relatively lower activity score (Fig. 4b). These results confirm a clear difference in computational *k*_cat_ values between missense and nonsense variants, highlighting the unique capability of DeepEnzyme to detect and discriminate these kinds of variations.

To further evaluate the generalization ability of DeepEnzyme in predicting *k*_cat_ values for enzyme variants from the saturation mutagenesis experiment, we utilized DeepEnzyme to predict *k*_cat_ values for large-scale mutants of phosphate-irrepressible alkaline phosphatase of Flavobacterium (PafA) [41]. Firstly, based on experimental measurement, all PafA mutations were categorized into two distinct categories: high *k*_cat_ mutations and low *k*_cat_ mutations, representing mutations with higher and lower experimentally-measured *k*_cat_ values compared to the wild-type enzyme (Fig. 4d). With DeepEnzyme, the median predicted *k*_cat_ values for the high *k*_cat_ mutations were observed to be 15% higher than those for the low *k*_cat_ mutation (*P* value = 0.0033) (Fig. 4c). Therefore, the result showcases the potential of DeepEnzyme in predicting the *k*_cat_ for enzyme variants from the saturation mutagenesis experiment.

### DeepEnzyme could identify key residue sites related to enzyme functionality

**Figure 5:**
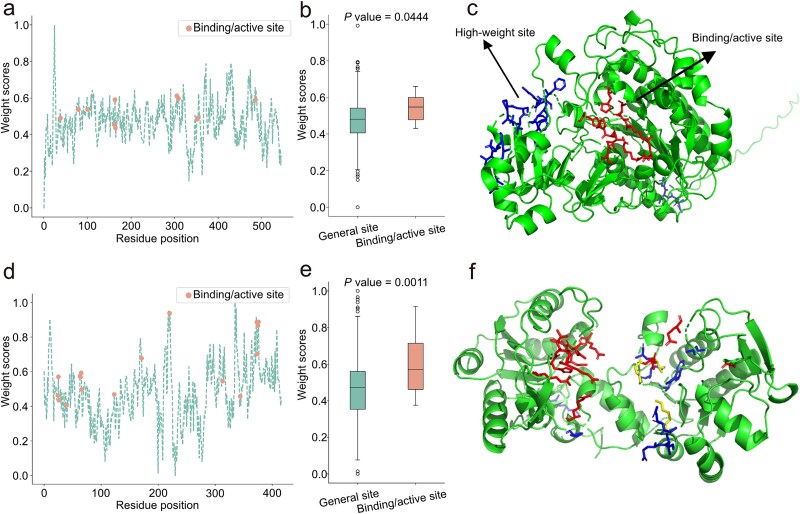
**Comparison between the binding/active site and high-weight site (these 5% residues sites with the highest weight scores calculated by DeepEnzyme) within protein 3D-structures.** (a) the weight scores of different residue sites in PafA; the red points are binding/active sites. (b) Comparison of the weight scores between the binding/active sites and general sites in PafA; the green box line indicates general sites, and the red box line indicates binding/active sites. (c) the distribution of the binding/active sites and high-weight sites regions within the 3D-structure of PafA, the red region represents for binding/active sites, the blue region for high-weight sites. (d) the weights of different residue sites in P00558; the red points are binding/active sites. (e) Comparison of the weights between the binding/active sites and general sites in P00558, where the green box line indicates general sites, and the red box line indicates binding/active sites. (f) the distribution of the binding/active sites and high-weight sites regions within the 3D-structure of P00558, the red region represents for binding/active sites, the blue region for high-weight sites, the yellow region for the overlap between the two.

The active site of an enzyme, characterized by specific amino acid residues in the protein structure, is pivotal for enzyme catalysis. In this context, we sought to investigate whether DeepEnzyme possesses the capability to identify the importance of active sites and binding sites within a protein 3D-structure. As the first example, we examined the case of PafA mentioned earlier. Leveraging site annotation data from the UniProt database, all residue sites in PafA were categorized into ‘binding/active sites’ and ‘general sites’. The structure vectors, which were computed by the protein structure baseline (Methods), followed by normalization using min-max normalization to obtain the weight score for each residue site. In addition to PafA, P00558 in the human EMP pathway was also used to validate DeepEnzyme performance. The results clearly displayed that the weight scores of binding/active sites in PafA and P00558 were notably higher than those of the general si (Fig. 5a, b, d, and e). At the same time, high-weight sites (These 5% residue sites with the highest weight scores) and binding/active sites in proteins are adjacent in structural space of PafA (Fig. 5c) or even have overlapping parts (Fig. 5f), and the percentage of hits was ~23.5% for P00558 (pLDDT = 96.58). Thus, DeepEnzyme showcased its potential in distinguishing and highlighting the biologically significant regions within protein 3D-structures, which may provide valuable clues for rational engineering of enzymes.

### DeepEnzyme accelerates the large-scale prediction of *k*_cat_ for enzymes from genome-scale metabolic models

**Figure 6:**
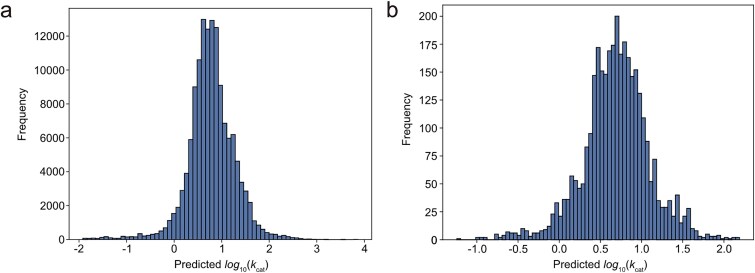
**Predicted *k*cat values for enzyme-catalyzed reactions in genome-scale metabolic models.** (a) Distribution of *k*cat values predicted by DeepEnzyme for enzyme-catalyzed reactions in metabolic models including those for *Homo sapiens*, *Mus musculus*, *Saccharomyces cerevisiae*, and *E. Coli* [42]. (b) Distribution of *k*cat values predicted by DeepEnzyme for enzyme-catalyzed reactions from the GEMs of *Geobacter metallireducens* GS-15 (BiGG ID: iAF987).

Genome-scale metabolic models (GEMs) are invaluable tools for characterizing cellular metabolism, often encompassing thousands of enzymes that catalyze various reactions. However, determining experimentally *k*_cat_ for a significant portion of enzymes in GEMs remains a challenging task. In this regard, DeepEnzyme presents itself as a convenient toolbox for predicting enzyme *k*_cat_ at the genome-scale. To illustrate the potential applications of DeepEnzyme, we gathered and processed GEMs from diverse organisms, including *E. coli*, *Mus musculus*, *Saccharomyces cerevisiae*, and *H. sapiens* [42], before *k*_cat_ prediction. Leveraging substrate and enzyme information, the genome-scale *k*_cat_ values could be predicted automatically (Fig. 6a and b). It shows that, at a holistic level, all *k*_cat_ values from multiple organisms or a single one exhibited a normal distribution, reflecting the obvious variance in predicted *k*_cat_ from different organisms.

## Discussion

Accurately predicting *k*_cat_ has become imperative for various applications, including protein engineering and enzyme design [3]. Herein, we present DeepEnzyme, an enhanced model to improve enzyme *k*_cat_ prediction by utilizing advanced deep learning architectures such as Transformer and GCN (Fig. 1). DeepEnzyme skillfully extracts and aggregates essential features from substrate, protein 1D sequences and 3D-structures. With significantly enhanced prediction accuracy, DeepEnzyme surpasses previously reported models in *k*_cat_ prediction by leveraging features from high-quality 3D-structures. Specifically, it achieves an impressively higher R^2^ value at ~0.6 on the test dataset (Fig.2 and Fig.3), outperforming TurNuP [14], DLKcat [13] and DLTKcat [33]. According to a latest study [43], when we prepared the manuscript, UniKP could outperform DeepEnzyme in prediction accuracy due to its usage of the pre-trained SMILES transformer model and protein language model. However, in a specific test, DeepEnzyme performs well in qualitatively predicting how single-point mutations determines the enzyme catalytic efficiency, thus showcasing the unique capability of DeepEnzyme (Supplementary Table 1). Meanwhile, with fewer parameters and smaller model size, DeepEnzyme is more than 2000 times faster than UniKP when completing the same prediction task, i.e. predicting *k*_cat_ for 7630 enzyme variants, assuming that the protein 3D-structures are ready in advance (testing with the UniKP code example on DELL Precision 5820 Tower).

Though not building on the pre-trained model, DeepEnzyme still shows reasonable robustness in predicting turnover numbers for remote enzyme homologs. The degree of similarity in protein sequences originated from the test and the training datasets could significantly influence the performance of deep learning models, and the prediction accuracy would decrease as the sequence dissimilarity increases [13, 14]. Through careful preprocessing of the sequence dataset, we significantly reduce the tendency of DeepEnzyme in overfitting during training, thereby improving the generalization ability of the model and ensuring the reliable *k*_cat_ predictions across diverse levels of sequence similarity. By comparison, even under circumstances with minimal sequence similarity (0–50%), DeepEnzyme exhibits exceptional prediction accuracy (Fig.3), thus successfully outperforming previous models [13, 14]. It is well known that protein 3D-structures are more evolutionarily conservative than amino acid sequences. Thus, extracting and utilizing the enzyme’s structural features during DeepEnzyme training enable the significant improvement in the accuracy of k*_cat_* prediction.

Furthermore, DeepEnzyme could help to evaluate of the influence of saturation mutagenesis on enzyme catalytic efficiency. In this aspect, the performance of DeepEnzyme was demonstrated using two enzymes. The results indicate that DeepEnzyme can, to a considerable extent, reflect how single mutations affect enzyme catalytic efficiency (Fig. 4). Moreover, DeepEnzyme assigns higher weight scores to active sites and binding sites within a protein 3D-structure. As these functional sites are closely correlated to enzyme function and activity [14] (Fig. 5), DeepEnzyme may empower the rational design of more efficient enzymes.

While DeepEnzyme achieves improved *k*_cat_ prediction accuracy compared to existing models, there is room for further enhancement in the following aspects. Firstly, the dataset used in this work encompassed less than 12 000 enzyme-substrate pairs. Expanding the dataset to encompass a more diverse enzyme-substrate pairs could further enhance the predictive performance of DeepEnzyme. Secondly, the pivotal experimental parameters such as pH and temperature were not taken into account during the model training in this work, which may contribute to discrepancies between predicted and measured *k*_cat_ values for certain enzymes. Additionally, multiple advanced pre-training models can be further merged into the current framework of DeepEnzyme to improve the prediction accuracy. It is envisioned that, together with other deep learning models, DeepEnzyme will undoubtedly provide new chances to characterize the internal correlations among 1D sequence, 3D-structures and enzyme catalytic efficiency, thus accelerating the protein engineering in the coming years.

## Methods

### Data preprocessing

In the reconstruction of DeepEnzyme, the DLKcat dataset was first downloaded [13]. In order to assess the similarity of enzyme sequence across the dataset, the MMseqs2 [34] was employed. To mitigate the impact of high sequence similarity among enzyme-substrate pairs in the dataset during model training, only the enzyme-substrate pairs with the highest enzyme sequence length among those with identical substrates and sequence similarity greater than 90% was retained. After data preprocessing, the dataset includes 11 927 distinct combinations of enzymes and substrates (Supplementary Table 2). Additionally, the dataset is randomly divided into groups of training, validation, and testing with ratio at 80%, 10%, and 10%.

### Enzyme structure prediction and contact map generation

In order to predict the enzyme structure and gather the structural data of enzymes, ColabFold [23] was used to predict protein structure, which is ~40–60 times faster than AlphaFold2, and the structures used in this work are of high quality (Fig. 1). Next, the protein 3D-structure was converted into a contact map, which could transform the protein structures into tensor format suitable for deep learning. For creating the contact map, the residues within the protein 3D-structures were defined as nodes, while two nodes were connected by a linker if the distance between the respective ${C}_{\alpha }$ atoms of two residues are smaller than $10\text{\AA} $ [44, 45].

### Deep learning pipeline for turnover number prediction in DeepEnzyme

The Transformer and GCN are particularly designed to extract hidden features from both protein 1D-sequence and 3D-structure. The model architecture and the pipeline for training and testing DeepEnzme are as the following.

*The protein sequence baseline.* In order to extract protein sequence features, we employ the Transformer model. Similar to the protein sequence processing in DLKcat, the protein sequence is split into an overlapping sequence composed by *n*-grams of amino acids. Before the Transformer, an embedding layer is employed to convert the protein sequence into a word tensor. The hidden vectors of the protein sequence are then determined using word embedding as input, followed by a Transformer encoder with position encodings (Eq.1), multi-head attention layers (Eq. 2, 3).


(1) \begin{equation*} {\displaystyle \begin{array}{l}{PE}_{pos,2i}=\sin \left(\frac{{pos}}{{{2i}_{10000}/ {{d}_{model}}}}\right)\\{PE}_{pos,2i+1}=\cos \left(\frac{{pos}}{{{2i}_{10000}/ {{d}_{model}}}}\right)\end{array}} \end{equation*}

(2) \begin{equation*} Attention\left(Q,K,V\right)= softmax\left(\frac{Q{K}^T}{\sqrt{d_k}}\right)V \end{equation*}

(3) \begin{equation*} {\displaystyle \begin{array}{l} MultiHead\left(Q,K,V\right)= Concat\left( hea{d}_1,\cdots, hea{d}_h\right){W}^o\\{} where\kern0.75em hea{d}_i= Attention\left(Q{W}_i^Q,K{W}_i^K,V{W}_i^V\right)\end{array}} \end{equation*}

where *pos* is the position of the amino acid, *d_model_* is the dimension of the vector, *Q, K,* and *V* stand for the vectors of Query, Key, and Value, respectively.

*The protein and substrate structure baseline.* The contact map is used to represent the protein structure, and the substrate graph is used to characterize substrate information. GCN generates substrate features and protein structure features as its output (Eq.4).


(4) \begin{equation*} {H}^{\left(l+1\right)}=\sigma \left({\overset{\sim }{D}}^{-\frac{1}{2}}\overset{\sim }{A}{\overset{\sim }{D}}^{-\frac{1}{2}}{H}^{(l)}{W}^{(l)}\right) \end{equation*}

*The neural attention mechanism baseline.* To obtain the final *k*_cat_ value output, the neural attention mechanism is incorporated, which enables us to capture the attention weights of various residue sites in the enzyme [46]. This attention mechanism (Eq. 5–7) works in conjunction with the Transformer and GCN components.


(5) \begin{equation*} \mathrm{C}=\left\{{c}_1^t,{c}_2^t,\cdots, {c}_n^t\right\} \end{equation*}

(6) \begin{equation*} {h}_{substrate}=f\left({W}_{inter}{y}_{substrate}+b\right) \end{equation*}

(7) \begin{equation*} {h}_i=f\left({W}_{inter}{c}_i+b\right) \end{equation*}

(8) \begin{equation*} {a}_i=\sigma \left({h}_{substrate}^T{h}_i\right) \end{equation*}

where $C$ is a set of hidden vectors representing the protein sequence, ${c}_1^t$ to ${c}_n^t$ are sub-hidden vectors for split subsequences within the protein sequence, ${y}_{substrate}$ is the molecular vector of the substrate, ${W}_{inter}$ and $b$ are the weight matrix and the bias vector, respectively, used in the neural network, $f$ is a nonlinear activation function (ReLU [47]), ${a}_i$ is the final attention weight value, $\sigma$ is the element-wise sigmoid function, and $T$ represents the transpose function.

*Evaluation metrics.* In this study, three performance metrics are used to evaluate the prediction performance of the model: the coefficient of determination (R^2^, Eq. 9), the root mean square error (RMSE, Eq. 10), and the Pearson correlation coefficient (PCC, Eq. 11), as calculated as the following.


(9) \begin{equation*} {R}^2=1-\frac{\sum \limits_{i=1}^n{\left({y}_{ie}-{y}_{ip}\right)}^2}{\sum \limits_{i=1}^n{\left({y}_{ie}-{\overline{y}}_{ie}\right)}^2}\qquad\qquad\qquad \end{equation*}

(10) \begin{equation*} RMSE=\sqrt{\frac{\sum \limits_{i=1}^n{\left({y}_{ie}-{y}_{ip}\right)}^2}{n}} \qquad\qquad\qquad\qquad\end{equation*}

(11) \begin{equation*} PCC=\frac{\sum \limits_{i=1}^n\left({y}_{ie}-{\overline{y}}_{ie}\right)\left({y}_{ip}-{\overline{y}}_{ip}\right)}{\sqrt{\sum \limits_{i=1}^n{\left({y}_{ie}-{\overline{y}}_{ie}\right)}^2}\sqrt{\sum \limits_{i=1}^n{\left({y}_{ip}-{\overline{y}}_{ip}\right)}^2}}, \end{equation*}

*where*  ${y}_{ie}$ represents the experimental value of *k*_cat_, ${y}_{ip}$ represents a predicted value for *k*_cat_, and $\overline{y}$ represents the corresponding averages.

### Comparison of different deep learning models in *k*_cat_ prediction

Utilizing the code and datasets in GitHub, the prediction performance of DLKcat [13] and TurNuP [14] was evaluated respectively. MMseqs2 [34] was utilized to determine the degree of similarity between the protein sequences in the training dataset and the test dataset, FoldSeek [35] and US-align [36] were used to determine the structural similarity of two proteins.

### Prediction performance of DeepEnzyme based on large-scale enzyme saturation mutagenesis experiments

Sequence data for CYP2C9 variants and PafA mutations was obtained from previous two studies [40, 41], respectively, and ColabFold was used to predict the 3D-structures for all variants of CYP2C9 and PafA. The *k*_cat_ values for mutant sequences were predicted directly with DeepEnzyme, which were then evaluated based on the experimental enzyme kinetic datasets from [40, 41].

### Interpretation analysis of DeepEnzyme in ranking the key residue sites related to enzyme activities

Wild-type PafA and P00558 were used as input for the *k*_cat_ prediction by DeepEnzyme, and the protein structural features were extracted by GCN, while min-max normalization was performed to obtain the weight of each residue site, and the sites were grouped according to the site classification in the UniProt database [8].

### Statistical analysis

In all statistical analysis, the two-sided t-test in the Python package SciPy was used [48].

**Key Points**

- DeepEnzyme was trained by leveraging the integrated features from both protein sequences and protein 3D-structures.
- DeepEnzyme exhibited remarkable robustness when processing enzymes with low sequence similarity compared to those in the training dataset.
- DeepEnzyme made it possible to evaluate how point mutations affect the catalytic activity of the enzyme.

## Supplementary Material

DeepEnzyme_file_Supplementary_bbae409

## Data Availability

The large file used to train the model could be found at the following link: https://figshare.com/articles/dataset/DeepEnzyme/25771062.

## Code availability

The codes are accessible at https://GitHub.com/hongzhonglu/DeepEnzyme.
