# Supplementary material for: DeepEnzyme: a robust deep learning model for improved enzyme turnover number prediction by utilizing features of protein 3D-structures
Source: Brief Bioinform. 2024 Aug 20;25(5):bbae409. doi: 10.1093/bib/bbae409 (PMC11880767; doi:10.1093/bib/bbae409)
Supplement: DeepEnzyme_file_Supplementary_bbae409 [file deepenzyme_file_supplementary_bbae409.docx]

**Supplementary file**

In order to further evaluate the predicted performance of DeepEnzyme, the experimentally measured *k*_cat_ values for tyrosine ammonia lyase from *Rhodotorula glutinis* (RgTAL) conducted by Yu et al[1] were collected. As reported, RgTAL functions as an essential enzyme in the phenylalanine metabolic pathway, performing crucial roles in biological systems[2, 3]. Yu et al. generated 13167 mutant sequences of RgTAL via single-point mutagenesis[1]. They then used UniKP to predict these mutants' *k*_cat_ and *k*_cat_ /*K*_m_ values. Following this, ten mutants were selected for experimental validation based on their highest predicted *k*_cat_ values and *k*_cat_ / *K*_m_ values. Using DeepEnzyme, the *k*_cat_ values for these ten mutants could be predicted, six of which were consistent with the experimental observation (Supplementary Table 1). Although there were no RgTAL enzyme-substrate combinations in our training dataset, the protein sequence similarity analysis using MMseqs2 revealed that their exist three sequences highly similar to the wild-type RgTAL sequence (pidentiy=99%) in the original DLKcat dataset utilized by UniKP. By contrast, the corresponding homolog sequence only appeared once in the DeepEnzyme dataset. Thus, it hints that DeepEnzyme performs well in qualitatively predicting the changes in enzyme *k*_cat_ induced by single-point mutations.

**Supplementary Table 1 Comparison between the predicted enzyme catalytic efficiency by UniKP and DeepEnzyme, respectively.** The experimental result is for mutated sequences of tyrosine ammonia lyase from *Rhodotorula glutinis* (RgTAL) conducted by Yu et al[1]. In all the comparison, the wild sequence of RgTAL was set as the reference.

| Mutation | Experimental *k*_cat_ | UniKP | DeepEnzyme |
| --- | --- | --- | --- |
| MT-613P | NA | **+** | **+** |
| MT-603P | **-** | **+** | **+** |
| MT-366H | **-** | **+** | **-** |
| MT-366W | **-** | **+** | **+** |
| MT-587V | **+** | **+** | **+** |
| MT-10Y | **+** | **+** | **+** |
| MT-337C | **-** | **+** | **+** |
| MT-668S | **-** | **+** | **-** |
| MT-489T | **+** | **+** | **+** |
| MT-337D | **-** | **+** | **-** |

+: single mutation improves enzyme catalytic efficiency compared to the wild sequence.

-: single mutation decreases enzyme catalytic efficiency compared to the wild sequence.

We de-emphasized the enzyme-substrate pairs in which the sequence similarity of the enzymes was higher than 90% in the case of the same catalytic substrate molecule and only kept the enzyme-substrate pairs with the longest sequence lengths in this case (Supplementary Table 2).

**Supplementary Table 2 Information of the dataset.**

| Enzyme-substrate pairs | Enzyme | Substrate |
| --- | --- | --- |
| 11927 | 6195 | 2672 |

After several model trainings to explore the best combination of hyperparameters, the final combination of hyperparameters is obtained (Supplementary Table 3).

**Supplementary Table 3 Hyperparameters** **of DeepEnzyme**

| Hyperparameters | Learning rate | *n*-grams | *r*-radius | Layers of GCN |
| --- | --- | --- | --- | --- |
| Value | 0.001 | 4 | 2 | 3 |

| Hyperparameters | Multi-Head Attention | Layers of Fully Connected | Embedding Dim | Dropout |
| --- | --- | --- | --- | --- |
| Value | 4 | 3 | 64 | 0.3 |

The predicted *k*_cat_ and experimental *k*_cat_ of PafA and P00558 in the text from different prediction models are shown in the following table (Supplementary Table 4). Note that all models were trained based on the log-transformation of *_k_*_cat_, so there exist difference between the predicted *k*_cat_ and measured *k*_cat_ for P00558.

**Supplementary Table 4 Comparison of predicted and experimental *k*_cat_ values**

| Enzyme | DeepEnzyme | DLKcat | TurNup | UniKP | Experimental *k*_cat_ |
| --- | --- | --- | --- | --- | --- |
| PafA | 4.54 | 1.91 | 21.14 | 6.94 | NaN |
| P00558 | 13.03 | 60.70 | 140.23 | 4.55 | 800 |

**Reference**

1. Yu H, Deng H, He J et al. UniKP: a unified framework for the prediction of enzyme kinetic parameters, Nature Communications 2023;14:8211.

2. Jendresen CB, Stahlhut SG, Li M et al. Highly Active and Specific Tyrosine Ammonia-Lyases from Diverse Origins Enable Enhanced Production of Aromatic Compounds in Bacteria and Saccharomyces cerevisiae, Applied and Environmental Microbiology 2015;81:4458-4476.

3. Zhou S, Liu P, Chen J et al. Characterization of mutants of a tyrosine ammonia-lyase from Rhodotorula glutinis, Applied microbiology and biotechnology 2016;100:10443-10452.
